# Suicide-related behaviors in older patients with new anti-epileptic drug use: data from the VA hospital system

**DOI:** 10.1186/1741-7015-8-4

**Published:** 2010-01-11

**Authors:** Anne C VanCott, Joyce A Cramer, Laurel A Copeland, John E Zeber, Michael A Steinman, Jeffrey J Dersh, Mark E Glickman, Eric M Mortensen, Megan E Amuan, Mary Jo Pugh

**Affiliations:** 1 VA Pittsburgh Healthcare System, Neurology Division, Pittsburgh, PA, USA; 2 University of Pittsburgh, Department of Neurology, Pittsburgh, PA, USA; 3 Yale University, Department of Psychiatry, New Haven, CT, USA; 4 Epilepsy Therapy Project, Orange, CT, USA; 5 Veterans Affairs HSR&D: South Texas Veterans Health Care System (VERDICT), San Antonio, TX, USA; 6 University of Texas Health Science Center at San Antonio, Department of Psychiatry, San Antonio, TX, USA; 7 San Francisco VA Medical Center, San Francisco, CA, USA; 8 Division of Geriatrics, University of California, San Francisco, San Francisco, CA, USA; 9 Center for Health Quality, Outcomes and Economic Research, Edith Nourse Rogers Memorial Hospital, Bedford, MA, USA; 10 Boston University School of Public Health, Boston, MA, USA; 11 University of Texas Health Science Center at San Antonio, Department of General Medicine, San Antonio, TX, USA

## Abstract

**Background:**

The U.S. Food and Drug Administration (FDA) recently linked antiepileptic drug (AED) exposure to suicide-related behaviors based on meta-analysis of randomized clinical trials. We examined the relationship between suicide-related behaviors and different AEDs in older veterans receiving new AED monotherapy from the Veterans Health Administration (VA), controlling for potential confounders.

**Methods:**

VA and Medicare databases were used to identify veterans 66 years and older, who received a) care from the VA between 1999 and 2004, and b) an incident AED (monotherapy) prescription. Previously validated ICD-9-CM codes were used to identify suicidal ideation or behavior (suicide-related behaviors cases), epilepsy, and other conditions previously associated with suicide-related behaviors. Each case was matched to controls based on prior history of suicide-related behaviors, year of AED prescription, and epilepsy status.

**Results:**

The strongest predictor of suicide-related behaviors (N = 64; Controls N = 768) based on conditional logistic regression analysis was affective disorder (depression, anxiety, or post-traumatic stress disorder (PTSD); Odds Ratio 4.42, 95% CI 2.30 to 8.49) diagnosed before AED treatment. Increased suicide-related behaviors were not associated with individual AEDs, including the most commonly prescribed AED in the US - phenytoin.

**Conclusion:**

Our extensive diagnostic and treatment data demonstrated that the strongest predictor of suicide-related behaviors for older patients newly treated with AED monotherapy was a previous diagnosis of affective disorder. Additional, research using a larger sample is needed to clearly determine the risk of suicide-related behaviors among less commonly used AEDs.

## Background

At the end of January 2008, the FDA issued an alert indicating that antiepileptic drug (AED) treatment is associated with increased risk for suicidal ideation, attempt and completion. This decision was based on a meta-analysis of suicidal ideation and behavior in placebo-controlled clinical studies of 11 antiepileptic drugs used in the treatment of epilepsy, psychiatric disorders and other conditions (including migraine and neuropathic pain) [1]. A higher percentage (0.43%) of patients receiving an AED had suicidal behavior and ideation compared to patients in *placebo *groups (0.22%). In addition, the relative risk for suicide-related behaviors was found to be highest in patients being treated for epilepsy compared to those being treated with AEDs for other medical conditions. No differences in risk were found among the 11 AEDs studied. The FDA recommended that healthcare professionals should closely monitor all patients who are currently taking or starting any AED for behavioral changes. Healthcare providers were instructed to inform patients, their families, and caregivers of the potential for an increase in the risk of suicide-related behaviors. The FDA also advised providers to balance the clinical need for these medications with the elevated risk for suicide-related behaviors.

Recent concern has been raised regarding the ability to make appropriate recommendations regarding AEDs in what has been termed a *data poor environment *[2]. Avorn [2] suggested that the AED-suicide-related behaviors controversy reveals the limits of ascertaining adverse events in randomized clinical trials (RCT) designed specifically to meet the requirements for FDA approval. Several limitations of the FDA analysis suggest that additional research is needed to better understand and interpret the findings before definitively concluding that all AEDs increase risk for suicidal behavior and ideation in all age groups.

Not only did the FDA analysis exclude the most commonly prescribed AED (phenytoin) used to treat epilepsy in the US, but many of the individuals in the exposed groups were on multiple AEDs, a factor recognized to increase risk of suicide-related behaviors [3]. Moreover, the extent to which RCT study participants reflect patients in clinical practice is not clear because study inclusion and exclusion criteria are variable and are not always available for analytic purposes. Based on assessments of previous clinical trials [4], it is unlikely that RCT patients represent those seen in clinical practice, especially with regard to older adults, because enrollment is frequently limited with respect to age, co-morbid conditions, and disease severity.

The goal of this study was to assess variation in suicide-related behaviors, as defined by Silverman et al [5], in a population not well represented by the data used for the FDA analysis--individuals 66 years and older with new exposure to AEDs-using data from the largest integrated health care system in the United States, the Department of Veterans Affairs, Veterans Health Administration (VA). As older individuals are: 1) at greater risk for a number of conditions for which these drugs are used (for example, epilepsy, depression, chronic pain), [6-8] 2) are under-represented in RCTs, 3) are more likely to be treated with older AEDs not used in RCTs [9] and 4) face an increased risk of suicide-related behaviors [10-12]. Our ability to control for concomitant psychiatric conditions provides important insight into the relationship between AED exposure and suicide-related behaviors.

## Methods

We used national VA pharmacy, administrative, and Medicare data to identify cases and controls for this study. Cases and controls were selected from the population of VA patients 66 years and older who received care from the VA between October 1, 1999 and September 30, 2004, and who had VA pharmacy data available for at least one year prior to the initial AED prescription. Individuals receiving a new prescription for AED monotherapy without a previous AED prescription were included in this analysis.

From the cohort of older VA patients who received a new AED monotherapy during the study period, we identified individuals with a diagnosis of suicidal behavior or ideation (hereafter *suicide-related behaviors*) using ICD-9-CM codes (V62.84 Suicidal ideation, 300.9 suicidal tendencies, E9499, E950-E958, E962.0, E980-E989) for individuals who remained on AEDs through the time of suicide attempt or ideation. Those with any suicide attempt or ideation after discontinuing AED were not considered in this study.

After identification of cases, we randomly matched the maximal number of controls to each case (N = 12) to increase power. Controls were matched to cases based on history of suicide-related behaviors prior to AED prescription, the year of first AED prescription, and diagnosis of epilepsy.

Descriptions of epilepsy diagnosis and other measures used in the conditional logistic regression model follow. Because VA privacy rules preclude publication of data where any cell has fewer than 11 individuals, we condensed categories for a number of variables (described below) but were still unable to report frequencies for gender or race due to small numbers of women and non-white suicide-related behaviors cases.

Epilepsy diagnosis was identified using an algorithm validated for use with national VA and Medicare diagnostic data in conjunction with VA pharmacy data [9]. Briefly, we restricted the cohort to those with at least one year of VA pharmacy and administrative data to assure a new diagnosis of epilepsy (ICD-9-CM codes epilepsy (345.XX) or convulsion (780.39) and a new prescription of an AED. Those with a first diagnosis of epilepsy and a subsequent AED within a year were identified as having new-onset epilepsy. This algorithm was found to have positive predictive value of 0.98.

### Demographic Characteristics

Age, gender and race were obtained from VA data; Medicare data were used to supplement missing values. Since older age has been associated with suicide-related behaviors [10] age was classified as 66 to 74, 75 years and older. Race was classified as white and nonwhite because white veterans have an increased risk of suicide-related behaviors [11].

### Other Clinical Characteristics

We included clinical characteristics that are associated with use of AEDs and suicide-related behaviors in order to minimize the potential for confounding by indication. If we found a significant impact of an AED that is commonly used for psychiatric conditions (for example, valproate, lamotrigine) or chronic pain (for example, gabapentin), but did not control for those conditions in the model, it is possible that the underlying condition being treated is the force behind the significant relationship rather than medication itself.

### Prior psychiatric comorbidity

Individuals with a history of depression are consistently found to be at increased risk for suicide, as are individuals with other mental health conditions such as bipolar disorder [12]. Because certain AEDs are more likely to be prescribed for individuals with psychiatric comorbidity (for example, valproate, carbamazepine, lamotrigine) [13], we used ICD-9-CM codes to identify patients with a diagnosis of depression (296.2-296.3, 311), anxiety (300.00, 300.02, 300.09), post-traumatic stress disorder ((PTSD); 309.81), bipolar disorder (296.0-296.1, 296.4-296.8), schizophrenia (295.x [excluding 295.5]), substance abuse (291, 292, 303-305 excluding 305.1) and other mental illness (290-312, excluding the codes listed above). We combined depression, anxiety and PTSD as affective disorders, and schizophrenia, other psychoses and bipolar disorder as serious mental illness per Blow and colleagues [14]. Chi-square analyses confirmed that relationships with suicide-related behaviors were similar among these conditions. Indicator variables for affective disorder, serious mental illness, substance abuse, and other mental illness were included in the model to determine the unique contribution of each type of psychiatric comorbidity.

Other physical conditions that may affect suicide-related behaviors in older individuals include chronic pain, dementia, and profound disease burden [12]. Chronic pain was defined using ICD-9-CM codes for conditions identified as being associated with persistent pain in the elderly by the American Geriatrics Society which includes conditions such as neuropathic pain [15]. Dementia was defined by ICD-9-CM codes which have been validated in VA databases [16]. Disease burden was quantified using a count of chronic physical disease states defined by Selim's physical comorbidity index (CI), developed for the veteran population to assess disease burden. The physical CI counts 30 chronic physical conditions including stroke, hypertension, diabetes, cardiovascular disease and peripheral vascular disease [17]. As analyses using the continuous variable were difficult to interpret, and no information appeared to be lost, we used a median split to create low (0 to 6 conditions) and high (≥ 7 conditions) comorbidity groups to aid in interpretation.

### Analysis

We provided descriptive statistics for cases and controls, and bivariate analyses comparing the groups. Gabapentin was selected as the comparator because patients with gabapentin exposure comprised over 75% of the cohort, and no other single AED was used by over 10% of the cohort. We then analyzed the simultaneous effect of prognostic factors of suicide-related behaviors using conditional logistic regression (stratified by case/control status), implemented via the SAS PHREG procedure (SAS 9.1, ^© ^2002-2003 SAS Institute, Carey, NC, USA). The final model was determined by performing a backward variable selection procedure in which all the candidate variables described above were included in the conditional logistic regression, and then the least significant variables were removed one at a time until only variables significant at *P *< 15 remained.

## Results

**Figure 1:**
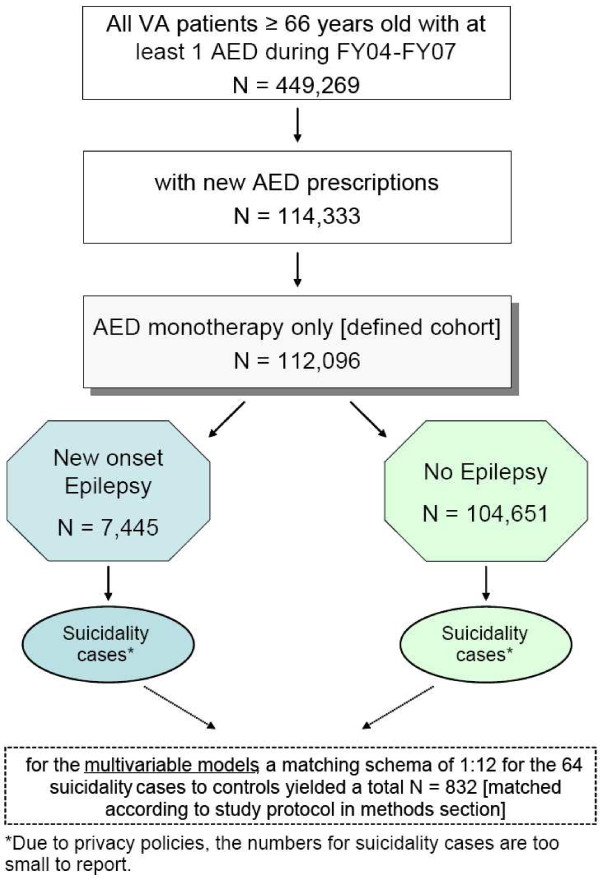
**Flow chart of study design and cohort definition**.

Of the 449,269 individuals who received an AED between FY 2000 and 2004, 114,333 had a new AED prescription. Figure 1 demonstrates that of the 112,096 (7,445 with new-onset epilepsy) who received AED monotherapy, 64 individuals had ICD-9-CM codes indicative of suicide-related behaviors. Rates of suicide-related behaviors were not significantly different for individuals with new-onset epilepsy and those with other diagnoses (*P *= 0.38). After matching 12 controls to each case based on prior history of suicide-related behaviors, epilepsy diagnosis and year of AED prescription, the case-control sample consisted of 832 individuals.

The sample was comprised primarily of men (97.5%, N = 811) and whites (86.4%, N = 719). Rates of suicide-related behaviors did not differ by gender (*P *= 0.61) or race (*P *= 0.58). Approximately half of the sample was 66 to 74 years of age (49.6%, N = 413), and there was no variation in suicide-related behaviors by age (42.2% (66 to 74 years) vs. 57.8% (75 years and older), chi square = 1.59; *P *= 0.45). This sample had high prevalence of chronic pain (85.7%, N = 713) prior to AED prescription; 12% had diagnosed dementia. The associations between suicide-related behaviors and chronic pain (*P *= 0.42) or chronic disease burden (*P *= 0.14) were not statistically significant, but diagnosis of dementia was significantly associated with suicide-related behaviors (42.2% with dementia vs. 25.8% without dementia; *P *< 0.01). Table 1 shows the prevalence of psychiatric comorbidity groups. The bivariate relationship between each psychiatric comorbidity group and suicide-related behaviors was statistically significant (*P *< 0.01).

**Table 1:** Psychiatric comorbidity groups among older veterans on new AED monotherapy

	Suicide-related behaviors Cases N = 64	Controls N = 768
**Psychiatric Conditions**	N (%)	N (%)

Affective Disorders^#^^	44 (68.8)	265 (34.5)

Serious Mental Illness*^#^	19 (29.7)	119 (15.5)

Substance Abuse/Dependence^^^	13 (20.3)	86 (11.2)

Other Mental Illness^^^	25 (39.1)	151 (19.7)

# Depression, Anxiety, PTSD

*Schizophrenia, Other Psychoses, Bipolar Disorder

^^^*P *< .001

Psychiatric comorbidity categories are not mutually exclusive; some patients had more than one concomitant psychiatric diagnosis.

Most individuals in this sample received AED prescriptions for gabapentin (76.8%; N = 639), followed by phenytoin (7.0%; N = 58), phenobarbital/primidone (6.6%; N = 55), valproate (5.9%; N = 49), and carbamazepine (3.1%, N = 24). The remainder received levetiracetam or lamotrigine (0.6%; N = 7). Because relationships with suicide-related behaviors for these drugs were similar and numbers for each drug were too low to examine individually, they were combined for this analysis. The bivariate relationship between type of AED and suicide-related behaviors was statistically significant, with more cases observed for valproate and lamotrigine/levetiracetam than expected by chance (*P *< 0.01). Conditional logistic regression models using backward elimination resulted in only two variables remaining in the final model: type of AED and affective disorders. Table 2 shows results of the final model. A trend for increased suicide-related behaviors among those prescribed levetiracetam or lamotrigine (Odds Ratio 10.2 95% CI 1.1 to 97.0) was found, but interpretation is difficult since few patients received either drug, and groups were combined in order to conduct the multivariable analysis. Individuals with a diagnosis of affective disorder prior to their prescription of AED were more likely to have subsequent suicide attempt or ideation than those without prior affective disorder (OR 4.2, 95% confidence interval 2.4 to 7.5).

**Table 2:** Conditional logistic regression model predicting suicide-related behaviors: psychiatric comorbidities and antiepileptic drugs.

	OR	95% Confidence Interval
Affective Disorders^#^	4.2	2.4 to 7.5

**Antiepileptic Drugs **(vs. Gabapentin)		

Phenobarbital	0.80	0.2 to 2.6

Phenytoin	1.0	0.2 to 4.9

Carbamazepine	1.2	0.3 to 5.5

Valproate	2.3	1.0 to 5.3

Newer AEDs	10.2	1.1 to 97.0

# Depression, Anxiety, PTSD

## Discussion

There has been significant concern regarding the extent to which findings from the FDA analysis of suicide-related behaviors in patients with AED exposure generalize to all patients receiving AEDs. Not all AEDs were included in the FDA analysis, yet all AEDs were identified as having increased risk of suicide-related behaviors. The assessment of risk was ascertained using data not designed to examine this risk, and using populations that are not necessarily similar to those in clinical practice [2,18]. The focus of our study allowed us to begin to address variation in risk for suicide-related behaviors among older patients receiving new AED monotherapy. Affective disorders are associated with suicide-related behaviors, and certain AEDs are more likely to be dispensed to persons with psychiatric comorbidities such as depression and bipolar disorder. An important contribution of this study was to control for psychiatric conditions that may be associated with suicide-related behaviors, and which may have been the reason for the AED prescription.

Our analysis found that the absolute risk of suicide-related behaviors (0.06% (64/112,096)) was nearly 10-fold lower that that observed in the FDA study (0.43% (120/27,863)) in our study population. Although the majority of veterans received gabapentin in our study, 13.6% (N = 113) were treated with phenytoin or a barbiturate providing new information about the older AEDs not possible in the FDA analysis of recent RCT. Once psychiatric conditions and potential confounding by indication were controlled, we found that individuals receiving lamotrigine or levetiracetam were more likely to have suicide-related behaviors diagnoses than individuals receiving gabapentin. Previous work has linked specific AEDs, including levetiracetam, to suicide-related behaviors [19-21]. Moreover, the FDA data found similar trends for lamotrigine and topiramate [1]. Given the small number of individuals with exposure to lamotrigine or levetiracetam at the time of this study, our study lacked power to identify true differences if they existed among these patients. Additional research using a longer study period and more patients on newer AEDs is needed to fully address this question.

In older VA patients who were started on AED monotherapy, the strongest reliable predictor for suicide-related behaviors was the diagnosis of an affective disorder (depression, anxiety, PTSD) prior to initiation of AED treatment. Although a seemingly intuitive finding, the significant effect of a prior affective disorder in our model is a strong result given the comparatively small sample size. Our finding linking affective disorder with suicide-related behaviors is consistent with earlier studies that found that affective illness, particularly depression, is the predominant psychopathology associated with suicide-related behaviors in later life [22,23]. While other psychiatric diagnoses, including bipolar disorder and schizophrenia, were also associated with suicide-related behaviors in bivariate analyses, they were no longer significant after controlling for affective disorders. This is likely due to the fact that 30% of the sample had diagnoses for two or more psychiatric comorbidity groups, and over 80% of those with multiple psychiatric conditions were diagnosed with an affective disorder. Affective disorders are commonly associated with epilepsy and individuals with epilepsy have been reported to have a higher risk of suicide, even after controlling for co-morbid psychiatric disease and sociodemographic factors [24-26].

Our study findings were also different than the FDA's alert that reported that the relative risk for suicidality was highest in patients being treated for epilepsy compared to those being treated for other medical conditions. In our findings the rates of suicidal behavior were not significantly different for individuals with new-onset epilepsy. However, our study included only those with new-onset epilepsy. Unlike prior research [27], our study results did not find a significant relationship between suicide-related behaviors and chronic pain or high disease burden. Moreover, the relationship between dementia and suicide-related behaviors was not significant after psychiatric comorbidities and AEDs were included in the multivariable model, suggesting that the relationship may be indirect through depressive disorders for both suicide-related behaviors and dementia.

There are several limitations of the current study. First, suicide-related behaviors were infrequent so the sample size was small, although considerably larger than the FDA analysis. The population studied were older adults treated with AED monotherapy, therefore these findings may not reflect the effects of AED in younger age groups and those treated with polytherapy. Due to the small number of individuals prescribed levetiracetam and lamotrigine, when analyzed individually an analysis could not generate a reliable odds ratio or confidence interval. Therefore the data from these two AEDs were combined. In the future, larger numbers of events might point to potential differences among various AEDs or different diagnostic groups. Second, the administrative data may have limited our ability to identify all cases of suicide-related behaviors. Our method is similar to studies by Valenstein and colleagues examining suicide-related behaviors in the VA, suggesting it is a reasonable approach [28,29]. Our use of both VA and Medicare data would identify individuals who received care in either VA or Medicare-reimbursed systems data, thus this limitation would affect all individuals on AEDs, thereby minimizing bias. Moreover, the study population consisted of older VA patients who were almost all men and predominantly white. Thus, findings may not generalize to younger patients or women. The racial composition of the sample was similar to the racial composition of older Americans [30]. Finally, because we do not have data for individuals not exposed to AEDs we can only examine differences among AEDs.

## Conclusions

The FDA alert has the potential to significantly change the medical management of a variety of medical conditions by altering healthcare providers' prescribing patterns and patient compliance. Following a review of the literature, Hesdorffer and Kanner recently concluded that AEDs probably have little impact on the relationship between suicide-related behaviors and epilepsy [31]. Most likely suicide-related behaviors in individuals treated with AEDs is multifactorial, including disease type/severity, AED type/dose/treatment duration and co-existing psychiatric conditions. Our study found that in older patients with new monotherapy AED use, the most important predictive factor of suicide was a diagnosis of affective disorder prior to initiation of AED monotherapy treatment. This is consistent with other studies addressing suicide in the elderly. One study estimated that 74% of late life suicides would be prevented if affective illness were eliminated from the population [32], illustrating the need for accurate diagnosis and treatment of affective disorders in the elderly. As our study found that prior psychiatric comorbidity was the strongest predictor of suicide-related behaviors in our sample of older individuals receiving new AED monotherapy, additional research of large clinical samples of all ages is needed before we can confirm the finding that AED exposure is associated with suicide-related behaviors in the general population of AED users, and not due to confounding.

## Abbreviations

AED: antiepileptic drug; FDA: Food and Drug Administration; RCT: Randomized Clinical Trial; US: United States; VA: Veterans Health Administration

## Competing interests

Anne C. Van Cott has research funding from GlaxoSmithKline. Joyce Cramer has been a consultant to Johnson and Johnson, Pfizer, Sepracor and UCB Pharma. Dr. Steinman is supported by a VA Career Development Transition Award (01-013) and a National Institute on Aging Paul Beeson Career Development Award (1K23AG030999). Dr. Steinman served as an unpaid expert witness for the plaintiff in United States ex. rel. Franklin vs. Pfizer, Inc, which alleged that Neurontin (gabapentin) was promoted for uses not approved by the FDA. Dr. Steinman also serves as co-investigator on an Attorneys General special grant funded with settlement monies from this litigation, and is a founding member of the Drug Industry Document Archive, seed money for which was provided by the plaintiff's lead lawyer in the aforementioned litigation.

The other authors report no conflicts of interest.

## Authors' contributions

ACVC assisted in the interpretation of data analysis and drafting the manuscript; JAC assisted in the conception and design and drafting the manuscript. LAC assisted in interpretation of data analysis and drafting the manuscript; JEZ assisted in the interpretation of data analysis and drafting the manuscript; and MAS assisted in the design of the analysis and drafting the manuscript. JJD assisted in the interpretation of data analysis and drafting the manuscript. MEG conceived the approach for statistical analysis and interpretation of the data analysis; EMM assisted in the analysis design and drafting the manuscript. MEA acquired the data for analysis, conducted the statistical analysis and assisted with drafting the manuscript. MJP acquired funding for the study, conceived the study design, acquired the data for analysis and assisted with interpretation of data analysis and manuscript preparation

## Pre-publication history

The pre-publication history for this paper can be accessed here:

http://www.biomedcentral.com/1741-7015/8/4/prepub
